# Use of chorionic gonadotropins during lactation to optimize postpartum sow reproductive performance: a review

**DOI:** 10.1590/1984-3143-AR2023-0118

**Published:** 2024-07-15

**Authors:** Monike Willemin Quirino, Carolini Schultz, Michele dos Passos Dezordi Franz, Thomaz Lucia, Arthur Martelli, Paulo Bayard Dias Gonçalves, Rafael da Rosa Ulguim, Bernardo Garziera Gasperin, Ivan Bianchi

**Affiliations:** 1 Mestrado Profissional em Produção e Sanidade Animal, Instituto Federal Catarinense, Araquari, SC, Brasil; 2 Núcleo de Pesquisa, Ensino e Extensão em Produção Animal, Instituto Federal Catarinense, Araquari, SC, Brasil; 3 Fisiopatologia e Biotécnicas da Reprodução Animal, Universidade Federal de Pelotas, Capão do Leão, RS, Brasil; 4 Universidade Federal de Santa Maria, Santa Maria, RS, Brasil; 5 Setor de Suínos, Universidade Federal do Rio Grande do Sul, Porto Alegre, RS, Brasil

**Keywords:** hormone therapy, lactation, estrus, ovulation, sows

## Abstract

Treating lactating sows with chorionic gonadotropins may allow controlling their post-weaning reproductive function, despite the occurrence of anestrous during lactation. This article reviews the potential effectiveness of treatment with both equine and human chorionic gonadotropins (eCG and hCG, respectively) during lactation on the control of estrus expression and ovulation in weaned sows. The use of 1,000 IU hCG at 24 and 48 h postpartum may induce ovulation in the treated sows, but the ovulation rate may be variable. Pregnancy rates may be improved with combined treatment after the second week of lactation with both chorionic gonadotropins: 1,500 IU eCG plus 500 – 1,000 hCG; or 1,000 IU eCG plus 1,000 IU hCG. Treatment with eCG (1,000 – 2,000 IU) at the end of lactation may result in acceptable estrus expression and ovulation rates, although with marginal benefit for pregnancy rates. The subsequent response to treatments with chorionic gonadotropins during lactation is likely influenced by the treatment period, the suckling frequency during lactation, and the boar exposure during the weaning-to-estrus interval. A better understanding of the efficiency of such steroid-free treatments is increasingly relevant due to the constraints of the use of steroid hormones in livestock reproductive management.

## Introduction

Pig production represents 34% of global meat consumption and the consumer demand for pork is expected to increase in the coming decades, thus, improving the efficiency of pork production is the most critical goal for producers (MacLeod et al., 2013; Kim et al., 2024). Reproductive efficiency is a key factor for the successful production of pork (Britt, 1986) and significant changes in the sector have occurred as a direct result of the use of reproductive technologies, such as artificial insemination (AI; Knox, 2016). In this regard, some strategies have been applied in commercial pig farms to optimize the AI efficiency and some related management factors (i.e., labor and facility efficiency), such as the use of hormonal therapies to control the female reproductive function (Wood et al., 1992).

Hormone therapies can control estrus and ovulation in both gilts and sows, especially considering the use of batch farrowing systems (Bown, 2006) and the possibility of using fixed-time AI in the future (reviewed by De Rensis and Kirkwood, 2016; Quirino et al., 2019). Several hormones can be used for these purposes, such as progestogens, prostaglandins, gonadotropin-releasing hormone (GnRH), and both hypophyseal and chorionic gonadotropins (reviewed by Knox, 2015; De Rensis and Kirkwood, 2016). The follicle-stimulating hormone (FSH) and the luteinizing hormone (LH) are hypophyseal gonadotropins naturally produced in both males and females from all species of the subphylum Vertebrata, whereas the equine (eCG) and human (hCG) chorionic gonadotropins are produced by the chorion of the placenta of some Equidae and primates, respectively (Hallast et al., 2008; Henke and Gromoll, 2008).

As hypophyseal and chorionic gonadotropins have high structural similarities and share common receptors (Campbell, 2005), hCG binds to LH receptors and eCG binds to both FSH and LH receptors (Senger, 2012). However, chorionic gonadotropins present a longer half-life than hypophyseal gonadotropins, due to the presence of a polysaccharide chain in their molecules. Additionally, chorionic gonadotropins have strong interspecies molecular homology (Henke and Gromoll, 2008), which allows their therapeutical use in other species, such as pigs. In this species, eCG and hCG are commonly associated to induce puberty in gilts and for treatment of postweaning anestrous in sows (reviewed by Estill, 2000; Innamma and Roongsitthichai, 2015).

Nevertheless, such hormones may be administered to lactating sows, which are in lactation-induced anestrous. That would suppress estrus expression after weaning in the treated sows to mitigate the consequences of early weaning or excessive catabolism (Zemitis et al., 2015; Kemp et al., 2018) and may also be potentially used to synchronize the postweaning estrus to homogenize the breeding groups, which is necessary in batch farrowing systems (Zemitis et al., 2015; Corezzolla et al., 2020). Furthermore, such treatment might allow AI during lactation, contributing to an increase in the number of litters weaned per female per year (Hausler et al., 1980).

Despite such potential benefits, treatment with chorionic gonadotropins in lactating sows is not commonly conducted in commercial pig farms. Nevertheless, the legislation of several countries restricts the use of steroid hormones to control the estrus cycle in livestock (European Union, 2003; Lane et al., 2008; FDA, 2021). Therefore, the use of chorionic gonadotropins may be a feasible alternative to the use of synthetic progestogens. The present review aimed to discuss the use of chorionic gonadotropins during the lactation of sows in therapies to control their subsequent reproductive function.

## Lactational anestrous

**Figure 1:**
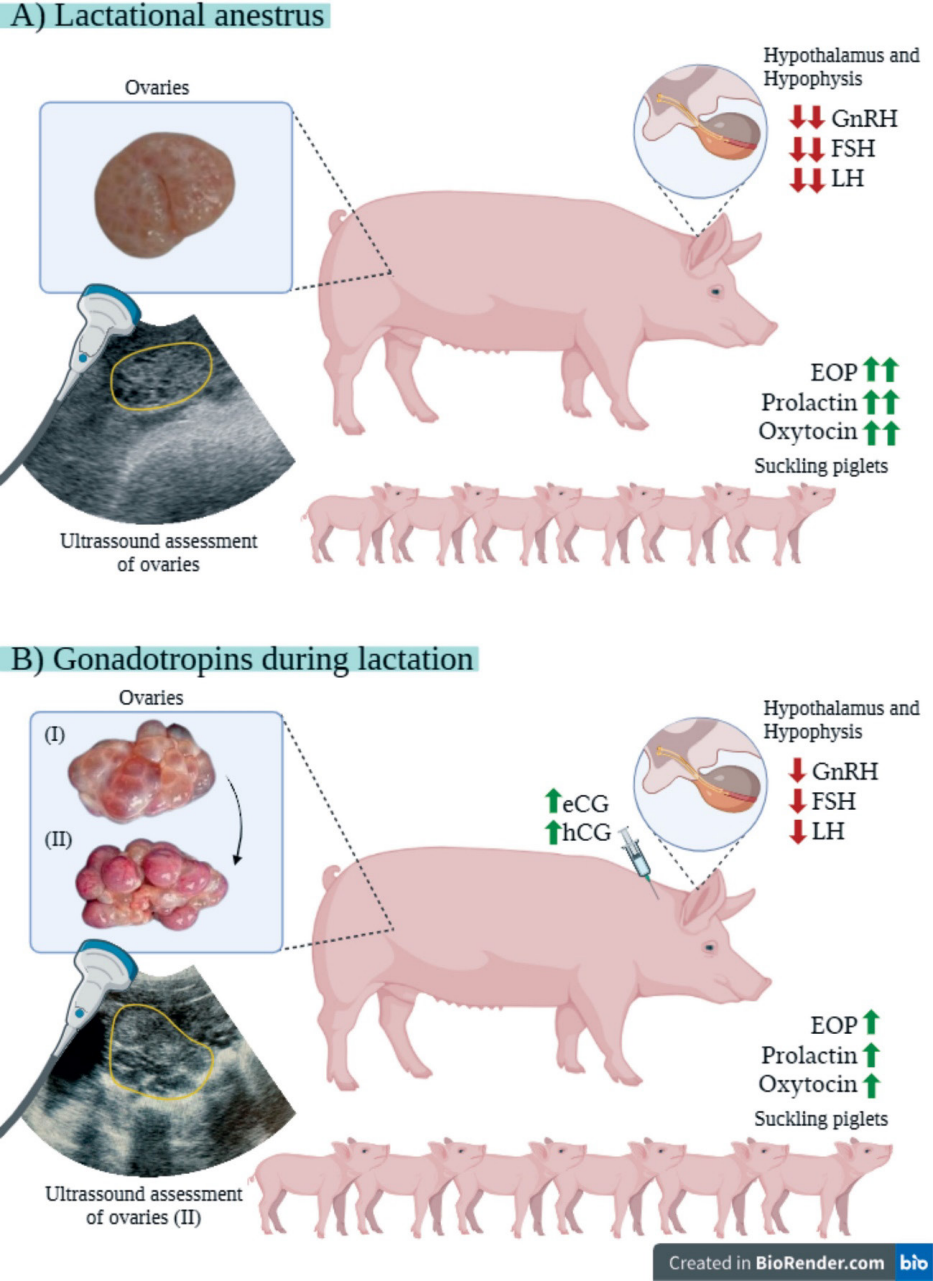
Scheme representing the lactational anestrus in sows **(A)** and the potential use of gonadotropins to induce follicular growth (I) and luteinization or ovulation (II) during this period for synchronizing the estrous cycle after weaning **(B**). GnRH: gonadotropin-releasing hormone; FSH: follicle-stimulating hormone; LH: luteinizing hormone; EOP: endogenous opioid peptides; eCG: equine chorionic gonadotropin; hCG: human chorionic gonadotropin.

The lactational anestrous is established from 6 h postpartum, when newborn piglets start suckling at regular intervals (De Rensis et al., 1993; Sesti and Britt, 1994), which promotes the release of hormones that stimulate lactogenesis and galactopoiesis, such as endogenous opioid peptides (EOP), oxytocin, prolactin, and somatotropin (Quesnel, 2009). Those substances inhibit GnRH release from the hypothalamus (Barb et al., 1994) and, thus, also inhibit LH and FSH release from the hypophysis (Wylot et al., 2013). As small and medium follicles with at most 2 mm diameter do not depend on gonadotropins for their development, they may be present in the ovaries of lactating sows but are unlikely to mature and ovulate, which would require highly frequent LH pulses (Quesnel, 2009). Consequently, while the suckling stimuli persist in the mammary glands, especially in large litters (Terry et al., 2014), lactating sows remain in anestrous (Costermans et al., 2020a; Figure 1A).

Among the EOP, dynorphins and enkephalins are the major suppressors of cyclicity in lactating sows (Barb et al., 1986). During lactation, the administration of morphine, an agonist of opioid receptors, is related to the reduction in serum LH concentration (De Rensis et al., 1998), whereas the administration of naloxone, an antagonist of EOP, is related to increased LH concentration, even though such effect is not observed in weaned sows (Barb et al., 1986; De Rensis et al., 1998). Oxytocin is also released through a neuroendocrine reflex induced by suckling. Besides its well-known effect on milk ejection, oxytocin is also related to behavioral and metabolic functions. Increased serum oxytocin concentration prepartum is associated with the nest-building behavior expressed by sows before farrowing (Yun et al., 2014). Oxytocin concentration is positively correlated with the concentration of non-esterified fatty acids (NEFA) and with weight loss in lactating sows (Valros et al., 2004). Additionally, oxytocin influences the secretion of prolactin and LH by porcine anterior pituitary cells *in vitro* (Bogacka et al., 2002).

The prolactin secretion is mostly mediated by the action of EOP (Armstrong et al., 1988). Prolactin concentration increases during the first two weeks of the lactation, but such levels gradually decline thereafter, as the suckling frequency is reduced (Schams et al., 1994). Nonetheless, contrary to what occurs in other mammals, in sows, the reduction in serum prolactin concentration is not followed by increased LH release during lactation (De Rensis et al., 1998). Even so, prolactin is involved in promoting lactational anestrous, since it acts directly at the ovarian level, modulating both steroidogenesis and angiogenesis, which are essential for follicle growth and development (Basini et al., 2014).

At low concentrations, prolactin is related to increased expression of FSH receptors in granulosa cells, indirectly stimulating progesterone and estradiol synthesis. On the other hand, at increased concentrations, prolactin inhibits the differentiation of granulosa cells and the expression of FSH receptors (Porter et al., 2000). The expression of genes that encode the prolactin molecule and receptors is detected in both theca and granulosa cells, indicating a paracrine effect of prolactin (Basini et al., 2014). Thus, although prolactin inhibits the synthesis of both progesterone and estradiol in granulosa cells in a dose-dependent mechanism, its stimulatory action over progesterone synthesis on theca cells and on luteal cells is even greater than that of LH (Słomczyńska et al., 2001). Additionally, prolactin presents a luteotropic effect during gestation (Ziecik et al., 2018), stimulating the expression of angiogenic factors *in vitro* (Basini et al., 2014).

The release of somatotropin (GH) is also stimulated by suckling (Lucy, 2008). Though, the release of both GH and the growth hormone-releasing factor (GHRF) is inhibited in the presence of raising naloxone concentrations, indicating that the somatotropic axis is regulated through the EOP during lactation (Armstrong et al., 1990). During lactation, the GH is related to increasing circulatory concentrations of insulin-like growth factor type 1 (IGF-1), NEFA, and glucose, which redirects the flow of energy and nutrients from the adipose tissue to the mammary gland (Lucy, 2008).

The neuroendocrine, metabolic, and hormonal stimuli triggered by suckling also redirect the sow’s energy reserves to milk production (Quesnel, 2009; Van Wettere et al., 2017). Therefore, the greater the number of suckling piglets, the greater both mammary gland development and milk production (Hansen et al., 2012), which commonly results in a negative energy balance, mainly when the feed consumption is inferior to the energy expense. Under such metabolic condition, signaling molecules such as IGF-1, insulin, and leptin are in reduced concentrations, indicating a decline in follicle growth, steroidogenesis, and oocyte quality (Costermans et al., 2020b; 2020c). Moreover, increased concentration of metabolites generated from muscle and fat catabolism, such as urea, creatine, and NEFA, are also related to reduced follicle development and steroidogenesis (Hoving et al., 2012; Costermans et al., 2020a).

## Post-weaning endocrine environment

As the lactation progresses, the negative feedback on the hypothalamus-hypophysis-ovaries axis is gradually weakened, as the piglets reduce the suckling frequency, start to explore the farrowing room environment, and begin to ingest a solid diet, which leads to a decline in milk production (Valros et al., 2002) and in protein and energy catabolism by sows (Hansen et al., 2012; Costermans et al., 2020a). That results in an increase in serum gonadotropin concentrations, especially of LH, which boosts follicle development (Lopes et al., 2020). Considering a common 3-week lactation, follicles exposed to this endocrine environment may reach 4 – 6 mm in diameter in the third week (Lucy et al., 2001).

After weaning, without local stimuli on the mammary glands, the LH pulses amplitude reduces while their frequency increases (Van den Brand et al., 2000), allowing the selection of responsive follicles previously recruited by the action of FSH (Quesnel, 2009), increased estradiol synthesis and subsequent estrus expression. Yet, the weaning-to-estrus interval (WEI) may be prolonged due to some risk factors. That may occur for sows with excessive catabolism during the lactation, in which an erratic pattern of LH release may impair follicle selection, resulting in irregular cyclicity and reduction in the subsequent litter size (Segura Correa et al., 2013; Rabelo et al., 2016). Those disorders may be frequent in primiparous sows, characterizing the second parity syndrome (Kemp et al., 2018), since primiparous sows may still need to achieve their adult weight and may have lower energy body reserves compared to multiparous sows (Hoving et al., 2012). Additionally, negative energy balance may be aggravated during periods of high environmental temperatures, in which the feed intake may be reduced (Rabelo et al., 2016), particularly in some hyper prolific genetic lineages (Hidalgo et al., 2014).

## Chorionic gonadotropins

The eCG and the hCG are heterodimer glycoproteins composed of two subunits: the α subunit is common to all gonadotropins; and the β subunit gives specific structural differences to each molecule (Campbell, 2005). The eCG starts to be synthesized by the trophoblastic cells of the endometrial calyces of the mare chorion approximately at the 38^th^ d of gestation. When administered to females from species other than equine, eCG acts as a gonadotropin. As the eCG molecule binds to FSH receptors, it stimulates follicle growth. Since eCG can also bind to LH receptors present in antral follicles, it may stimulate ovulation and help to luteinize follicles that eventually do not ovulate (Senger, 2012). Hence, eCG treatment is used to induce estrus and ovulation and to indirectly promote increased progesterone circulating concentration through the formation of accessory corpora lutea (Murphy, 2018).

The hCG is synthesized by syncytiotrophoblast cells of the human placenta since early gestation (Cole, 2010), also acting as a gonadotropin when used therapeutically, especially in treatments of reproductive disorders in women (Ezcurra and Humaidan, 2014) as well in animals (Am-in et al., 2018). The hCG can bind to LH receptors in the follicles and presents a longer half-life than LH (28 h vs 20 min). Thus, compared to LH, the actions of hCG on promoting ovulation, luteinization, and progesterone synthesis by corpora lutea are more intense (Ziecik et al., 2021).

Since both chorionic gonadotropins can promote follicle development and induce ovulation, their use in pigs is mostly aimed to induce puberty in gilts and to mitigate post-weaning anestrous in sows (reviewed by Estill, 2000; Innamma and Roongsitthichai, 2015). Such hormones may be also efficient when administered during lactation, to either suppress or synchronize estrus after weaning as well as to optimize AI programs in lactating sows.

### Estrus suppression and synchronization after weaning

Eventually, performing AI on the first estrus after weaning may be related to negative effects on the subsequent reproductive performance, resulting in increased embryo resorption rate and reduction in farrowing rate and litter size, as occurs when lactational catabolism is excessive, or after early weaning at periods shorter than 21 d (Levis, 1997; Zemitis et al., 2015). That may be avoided by skipping the first post-weaning estrus, allowing weaned sows to have more time to restore their body condition, and conducting AI at the second estrus after weaning (Hidalgo et al., 2014; Kemp et al., 2018). Nonetheless, the number of non-productive days added by such practice would correspond to the duration of an additional estrous cycle, which would impair the herd's reproductive efficiency (Dial et al., 1992). Thus, using chorionic gonadotropins during lactation may be an alternative to suppress the first post-weaning estrus for a shorter period, allowing the synchronization of the subsequent estrus (Kirkwood et al., 1999; Zemitis et al., 2015; Figure 1B). That would be particularly suitable for herds transitioning from conventional weekly management to batch farrowing (Corezzolla et al., 2020), optimizing labor and facility efficiency.

However, treatment with chorionic gonadotropins during lactation may result in inconsistent ovulation response (Table 1). Administration of 1,000 IU hCG to sows within 24 h after farrowing resulted in ovulation in 80% of primiparous and 71.9% of multiparous (Armstrong et al., 1999). In other studies (Kirkwood et al., 1999; Zemitis et al., 2015), the same treatment performed within 24 – 48 h postpartum resulted in lower ovulation rates (22 – 41%) in primiparous or multiparous sows. Additionally, treatment with the 400 IU eCG + 200 IU hCG combination 24 h postpartum was effective to induce the luteinization of antral follicles in females with parity ≥ 1 (Van Wettere et al., 2013). Although the reasons related to such discrepancies are not yet clearly established, the treatment period and the size of the available follicles are potential candidates (Armstrong et al., 1999; Kirkwood et al., 1999).

**Table 1:** Ovulation rate for sows treated with chorionic gonadotropins during lactation.

**Reference**	**Genetic**	**Parity**	**Protocol**	**Administration time** *	**Ovulation rate,** **% (n/n)**
Armstrong et al. (1999)	LW × LD × YS	1	1,000 IU hCG	24 h	80.0 (12/15)
		≥ 2	1,000 IU hCG	24 h	71.4 (15/21)
Kirkwood et al. (1999)	NI	≥ 1	1,000 IU hCG	24 h	40.6 (28/69)
Zemitis et al. (2015)	NI	≥ 1	1,000 IU hCG	24 h	31.3 (5/16)
		≥ 1	1,000 IU hCG	48 h	22.2 (4/18)
Van Wettere et al. (2013)	LW × LD	≥ 1	400 IU eCG + 200 IU hCG	24 h	5.6 (1/18)

* Post-partum. hCG: human chorionic gonadotropin; eCG: equine chorionic gonadotropin. LW: Large White; LD: Landrace; YS: Yorkshire; NI: Not informed.

For the primiparous and multiparous sows that ovulated after hCG treatment 24 h post-farrowing and weaned after 14 and 18 d, the average WEI was 10 d. Furthermore, when prostaglandin F2_α_ (PGF2_α_) was administered at weaning (14 d postpartum), the WEI was prolonged up to 17 d (Armstrong et al., 1999). In fact, lactogenic hormones released during lactation, such as prolactin, somatotropin, and IGF-1, may also have luteotropic action, stimulating progesterone synthesis from corpora lutea during lactation (Yuan and Lucy, 1996). That suggests that the corpora lutea formed during lactation may last longer than those formed during regular estrous cycles, which may explain why sows weaned after either 14 or 18 d of lactation presented similar WEI (Armstrong et al., 1999). Though, depending on the period of the estrous cycle, administration of exogenous PGF2_α_ may either inhibit or stimulate progesterone synthesis by corpora lutea, as observed *in vitro* (Przygrodzka et al., 2014).

In a natural estrous cycle, before the 12^th^ d, the PGF2_α_ binds to receptors on luteal cells and acts synergically with LH and prostaglandin E2 to activate cyclic adenosine monophosphate (cAMP), stimulating steroidogenesis and angiogenesis (Ziecik et al., 2018). Subsequently, after 12 d, corpora lutea acquire luteolytic capacity, since PGF2_α_ triggers transcriptional changes through activation of inositol triphosphate (IP3), which down-regulates genes that code LH receptors and the StAR protein (Diaz and Wiltbank, 2005), stimulating luteolysis (Ziecik et al., 2018). Therefore, the similar WEI observed for primiparous and multiparous sows weaned after hCG treatment either at 14 or 18 d during lactation (Armstrong et al., 1999) may be because PGF2_α_ was administered before the acquisition of luteolytic activity by the corpora lutea.

It is important to consider that luteinization/ovulation may occur later after hCG treatment in early postpartum sows compared to weaned or cyclic females, since luteinized follicles could not be identified through ultrasonography 3 d after hCG treatment in sows with parity ≥ 1 treated with hCG 24 or 48 h post-partum (Zemitis et al., 2015). As follicles having 3 – 4 mm diameter grow naturally following LH stimulus (Driancourt et al., 1995), ovulations induced by hCG treatment in lactating sows may be spontaneous, after the endogenous LH increase as lactation progresses, or directly related to hCG treatment, due to its long half-life. Also, lactogenic hormones may influence the acquisition of luteolytic capacity by the corpora lutea explaining, at least in part, the refractoriness to PGF2_α_ treatment at weaning in sows previously treated with hCG, which still needs to be investigated.

The administration of eCG during lactation can also be used to induce ovulation (Table 2). There is evidence that the closer eCG treatment is to the end of lactation, the greater would be the frequency of sows expressing estrus after weaning. According to Martinat-Botte (1975), administration of 2,000 IU eCG on the 16^th^ d of lactation results in estrus expression 3 to 7 d after treatment in nearly one-third of the treated sows. Post-weaning ovulation rates may be increased by combining the eCG treatment with farrowing room management practices aimed to stimulate LH release by reducing the number of suckling piglets (e.g., through intermittent or segregated suckling), which may result in estrus expression on more than 70% of the treated primiparous or multiparous sows (Cole and Hughes, 1946; Crighton, 1970). However, even though the observed rates of estrus expression and ovulation may be eventually similar (Martinat-Botte, 1975), some sows may ovulate without expressing estrous signs, especially when not exposed to contact with a boar, since the EOP may inhibit the expression of behavioral estrous signs during lactation (Fuentes-Hernández et al., 2011).

**Table 2:** Estrus expression and ovulation rate for sows treated with equine chorionic gonadotropin (eCG) during lactation.

**Reference**	**Genetic**	**Parity**	**Protocol**	**Administration time**	**Estrus expression, % (n/n)**	**Ovulation rate, %**
Cole and Hughes (1946)	PC, B and DJ	≥ 2	700 – 1,400 IU^*^	D0 – D37†	26.7% (4/15)	.
	PC, B and DJ	≥ 2	750 – 1,500 IU*	D38 – D67‡	96.3 (26/27)	.
Kirkwood and Thacker (1998)	YS × LD	NI	1,000 IU	D28**	85.2 (23/27)	92.6
Crighton (1970)	LW and LW × LD	≥ 1	1,500 IU	D23^**^	88.9 (16/18)	.
	LW and LW × LD	≥ 1	1,500 IU	D23^**^	77.3 (17/22)	.
Martinat-Botte (1975)	LW and PT	NI	2,000 IU	D16	33.3 (7/21)	.
	LW and PT	NI	2,000 IU	D16^**^	76.5 (13/17)	71.4
	LW and PT	NI	2,000 IU	D18	57.1 (117/205)	41.6
	LW and PT	NI	2,000 IU	D25	74.3 (104/140)	75.0

D0 = Day of farrowing; PC: Poland China; B: Berkshire; DJ: Duroc Jersey; YS: Yorkshire; LW: Large White; LD: Landrace; PT: Pietrain; NI: not informed. ^*^Dosages varying from 700 to 1,400 IU eCG or 750 to 1,500 IU eCG; ^†^Administration moment ranging from immediately postpartum to the 37^th^ d postpartum; ^‡^Administration moment ranging from the 38^th^ to the 67^th^ d postpartum; ^**^Concurrently reduction of suckling stimulus.

It is also important to mention that, regardless of the gonadotropin used, estrous detection conducted in the presence of a boar may by itself be a confounding factor. A positive response to boar exposure during lactation, when gonadotropin treatment is not used, may result in estrus expression within 5 d in more than 80% of the exposed sows, with subsequent ovulation in more than 60% of the sows with parity ≥ 1 (Van Wettere et al., 2013). The mechanism explaining the occurrence of LH release after boar exposure without exogenous hormone administration is not known, although it may involve the action of EOP, which may be elucidated through future research.

### Use of gonadotropin treatment to allow AI during lactation

Conducting AI in lactating sows may increase the number of litters per female per year (Hausler et al., 1980), which may boost the number of piglets weaned per female per year (Dial et al., 1992), as conception during lactation would allow delayed weaning, with no negative impact on overall herd productivity (Van Wettere et al., 2017). Piglets with older weaning age (e.g., 25 d) will be heavier when entering the nursery, present better immunity and gastrointestinal development, less incidence of diarrhea, and improved growth efficiency (López-Vergé et al., 2019; Ming et al., 2021). That would be financially beneficial for commercial farms, increasing the number of marketed pigs, due to a reduction of pig losses in the nursery, growing, and finishing phases (Faccin et al., 2020).

The occurrence of lactational anestrous may limit the adoption of AI during lactation, which would justify the use of therapies with chorionic gonadotropins to improve follicular growth, estrous expression, and AI in lactating sows, in the attempt to shorten the farrowing interval (Crighton, 1970; Hausler et al., 1980; Hodson et al., 1981). One of the first studies conducted in this field used different dosages of eCG (750 – 1500 IU) at various stages of lactation, and the authors reported that only 6% of the treated multiparous sows became pregnant when the eCG injection was performed between the farrowing and the 37^th^ d of lactation. When the hormone was administered between the 38^th^ and 67^th^ d of lactation, a pregnancy rate of 70.4% was observed (Cole and Hughes, 1946). Pregnancy rates near 60% were found for sows with parity order ≥ 1 when eCG was administered within 21 – 28 d of lactation (Crighton, 1970; Kirkwood and Thacker, 1998).

Greater pregnancy rates in primiparous and multiparous sows were observed combining eCG and hCG (Table 3), especially at increased doses: 1,000 – 1,500 IU eCG; and 500 – 1,000 IU hCG (Hodson et al., 1981). As the half-life of hCG is nearly 84-fold greater compared to LH, its luteotropic action results in substantial progestogen production (Ziecik et al., 2021), which stimulates histotrophic synthesis, favoring embryo nutrition and placenta formation (Almeida and Dias, 2022). Additionally, such treatment may mitigate seasonal negative effects on pregnancy rates, which are more pronounced in periods of high temperature (Hodson et al., 1981).

**Table 3:** Pregnancy rate for sows treated with equine chorionic gonadotropin (eCG) – alone or combined with human chorionic gonadotropin (hCG) – during lactation.

**Reference**	**Genetic**	**Parity**	**Protocol**	**Administration time**	**Breeding**	**Pregnancy rate, % (n/n)**
Cole and Hughes (1946)	PC, B and DJ	≥ 2	700 – 1,400 IU eCG*	D0 – D37†	NM	6.7 (1/15)
	PC, B and DJ	≥ 2	750 – 1,500 IU eCG^*^	D38 – D67‡	NM	70.4 (19/27)
Crighton (1970)	LW and LW × LD	≥ 1	1,500 IU eCG	D23^**^	NM	61.1 (11/18)
	LW and LW × LD	≥ 1	1,500 IU eCG	D23^**^	NM	59.1 (13/22)
Kirkwood and Thacker (1998)	YS × LD	NI	1,000 IU eCG	D28**	NM	65.2 (15/23)
Hausler et al. (1980)	DR × HS × YS	≥ 1	1,500 IU eCG + 1,000 IU hCG	D13 – D32§	AI	80.0 (12/15)
	DR × HS × YS	≥ 1	1,500 IU eCG + 1,000 IU hCG	D5	AI	0.0 (0/10)
	DR × HS × YS	≥ 1	1,500 IU eCG + 1,000 IU hCG	D10	AI	20.0 (2/10)
	DR × HS × YS	≥ 1	1,500 IU eCG + 1,000 IU hCG	D15	AI	80.0 (8/10)
	DR × HS × YS	≥ 1	1,500 IU eCG + 1,000 IU hCG	D20	AI	60.0 (6/10)
Hodson et al. (1981)	NI	≥ 1	1,500 IU eCG +1,000 IU hCG	D16 – D37#	AI	80.0 (12/15)
	NI	≥ 1	1,500 IU eCG + 500 IU hCG	D16 – D37^#^	AI	60.0 (9/15)
	NI	≥ 1	1,000 IU eCG + 1,000 IU hCG	D16 – D37^#^	AI	71.4 (10/14)
	NI	≥ 1	1,000 IU eCG + 500 IU hCG	D16 – D37^#^	AI	46.7 (7/15)

D0 = Day of farrowing; PC: Poland China; B: Berkshire; DJ: Duroc Jersey; LW: Large White; LD: Landrace; DR: Duroc; HS: Hampshire; YS: Yorkshire; NI: not informed; NM: natural mating; AI: artificial insemination. ^*^Dosages varying from 700 to 1,400 IU eCG or 750 to 1,500 IU eCG; ^†^Administration moment ranging from immediately postpartum to the 37^th^ d postpartum; ^‡^Administration moment ranging from the 38^th^ to the 67^th^ d postpartum; **Concurrently reduction of suckling stimulus; ^§^Administration moment ranging from the 13^th^ to the 32^th^ d postpartum; ^#^Administration moment ranging from the 16^th^ to the 37^th^ d postpartum.

Nevertheless, pregnancy rates observed after administration of high doses of eCG and hCG within a 96 h-interval on different days of lactation presented inconsistent results (Hausler et al., 1980). Those findings suggest that the uterine environment is not suitable to support conception and embryo development before the second week postpartum, during which uterine involution commonly occurs (Meile et al., 2020). Therefore, compared to sows inseminated during such period, those inseminated within 23 – 25 d postpartum achieve greater farrowing rates (Levis, 1997). Treatments combining eCG and hCG can be applied during consecutive lactations, with satisfactory pregnancy rates and no relevant production of anti-eCG antibodies (Crighton, 1970; Hodson et al., 1981).

## Progestogens during lactation

Suppression and synchronization of post-weaning estrus can also be accomplished through supplementation with progestogens during lactation. Compared to non-supplemented sows (primiparous and multiparous), those supplemented with altrenogest from the 12^th^ to the 18^th^ d of lactation and weaned at the 21^st^ d presented a similar number of follicles and farrowing rate, but greater follicle diameter at the time of estrus and greater subsequent litter size (Lopes et al., 2017). When the same supplementation was conducted in primiparous and multiparous sows during the last week of a 3-week lactation, follicle diameter at weaning was increased, corpora lutea were larger and uniform and estrus expression was concentrated 5 d after weaning (Gianluppi et al., 2021).

Despite those promising results, it is important to emphasize that altrenogest, the only progestogen commercially available for use in sows, may be considered costly and labor-intensive since it requires oral supplementation every 24 h (Haas et al., 2017). Intravaginal devices for slow progestogen release, efficiently used to control estrous cycle in ruminants, have been tested in swine by our group (Gasperin et al., 2011; Freling et al., 2013; Ulguim et al., 2019; Quirino et al., 2020; Fiúza et al., 2023), but such devices are still not validated for this species and currently not available in the market. Based on the effectiveness of gonadotropins treatment during lactation in inducing ovulation and luteinization, eCG and/or hCG treatment may represent an alternative to synthetic progestagens, which is currently being investigated by our group.

The need for alternative protocols to control the estrous cycle of sows is also justified by the restrictions to the use of steroid hormones imposed by some markets, such as the European Union, New Zealand, Australia, USA, and Canada (European Union, 2003; Lane et al., 2008; FDA, 2021). Such restrictions are based upon concerns related to potential environmental contamination and the negative collateral effects of residues of such hormones (Karg and Vogt, 1978).

## Final considerations

Despite the occurrence of lactational anestrous, treatment with chorionic gonadotropins in lactating sows can be an important management strategy to control the subsequent reproductive cyclicity. Treatment with 1,000 IU hCG at 24 and 48 h after farrowing can induce ovulation, whereas treatment with 1,000-2,000 IU eCG at the end of lactation may induce estrus and ovulation in a large frequency of treated sows. Such therapies can be used to either suppress or synchronize estrous after weaning, also allowing AI to be conducted during lactation. Improvement in pregnancy rates can be achieved through the administration of high doses of chorionic gonadotropins (e.g., 1,500 IU eCG plus 500 – 1,000 IU hCG; or 1,000 IU eCG plus 1,000 IU hCG) after the third week of lactation. Future studies should evaluate whether the efficiency of such treatments may be influenced by factors such as the reduced suckling stimuli in farrowing rooms and the presence of boars during estrus detection. Furthermore, the viability of using such an approach as an alternative to synthetic steroid treatment to prolong the WEI deserves investigation.
